# Change Point Test for the Conditional Mean of Time Series of Counts Based on Support Vector Regression

**DOI:** 10.3390/e23040433

**Published:** 2021-04-07

**Authors:** Sangyeol Lee, Sangjo Lee

**Affiliations:** Department of Statistics, Seoul National University, Seoul 08826, Korea; lscho13@snu.ac.kr

**Keywords:** time series of counts, INGARCH model, SVR and TSVR with PSO, change point detection, CUSUM test

## Abstract

This study considers support vector regression (SVR) and twin SVR (TSVR) for the time series of counts, wherein the hyper parameters are tuned using the particle swarm optimization (PSO) method. For prediction, we employ the framework of integer-valued generalized autoregressive conditional heteroskedasticity (INGARCH) models. As an application, we consider change point problems, using the cumulative sum (CUSUM) test based on the residuals obtained from the PSO-SVR and PSO-TSVR methods. We conduct Monte Carlo simulation experiments to illustrate the methods’ validity with various linear and nonlinear INGARCH models. Subsequently, a real data analysis, with the return times of extreme events constructed based on the daily log-returns of Goldman Sachs stock prices, is conducted to exhibit its scope of application.

## 1. Introduction

In this study, we developed a forecasting method for the time series of counts based on support vector regression (SVR) with particle swarm optimization (PSO), and used it to detect a change in the conditional mean of the time series based on the cumulative sum (CUSUM) test that is calculated from integer-valued autoregressive conditional heteroscedastic (INGARCH) residuals. Over the past few decades, the time series of counts have gained increased attention from researchers in diverse scientific areas. Considering the research conducted by [1,2,3,4,5], two classes of models, such as integer-valued autoregressive (INAR) and INGARCH models, have been popular for analyzing the time series of counts. See [6] for more details. These models have been harnessed to analyze polio data [7], crime data [8], car accident traffic data [9], and financial data [10].

Although the basic theories and analytical tools for these models are quite well developed in the literature, as seen in [11,12,13,14,15], a restriction on their usage exists because both INAR and INGARCH models are mostly assumed to have a linear structure in their conditional mean. In INGARCH models, Poisson and negative binomial distributions have been widely adopted as the conditional distribution of current observations over past information. This is because assuming these distributions is not impractical, as the correct specification of underlying distributions is not essential when attempting to estimate the conditional mean equation, as demonstrated by [16], who considered the quasi-maximum likelihood estimation (QMLE) method for the time series of counts. However, for the QMLE approach to perform adequately, the conditional mean structure must be correctly specified. As misspecification can potentially lead to false conclusions in real situations, we considered SVR as a nonparametric algorithm for forecasting the time series of counts. To our knowledge, our current study is the first attempt in the literature to use SVR for the prediction of time series of counts based on the INGARCH scheme.

SVR has been one of the most popular nonparametric algorithms for forecasting time series and has been proven to outperform classical time series models, such as autoregressive and moving average (ARMA) and GARCH models, as SVR can approximate nonlinearity without knowing the underlying dynamic structure of time series [17,18,19,20,21,22,23,24,25]. SVR has the merit of implementing the “structural risk minimization principle” [26] and seeks a balance between model complexity and empirical risk [27]. Moreover, a smaller number of tuning parameters is required, and determining a global solution is not problematic because it solves a quadratic programming problem (QPP).

SVR has been modified in various manners; for example, smooth SVR [28], least squares (LS)-SVM [29], and twin SVR (TSVR) [30]. TSVR generates two hyperplanes unlike SVR and has a significant advantage over SVR in computational speed. For the relevant references, see [31,32,33]. Here, we harness the SVR and TSVR methods particularly with the particle swarm optimization (PSO) algorithm, originally proposed by [34], in determining a set of optimal parameters to enhance their efficacy. For an overview of PSO, see [35,36].

As an application of our SVR method, we consider the problem of detecting a significant change in the conditional mean of the INGARCH time series. Since [37], the parameter change detection problem has been a core issue in various research areas. As financial time series often suffer from structural changes, owing to changes in governmental policy and critical social events, and ignoring them leads to a false conclusion, change point tests have been considered as an important research topic in time series analysis. See [38,39] for a general review. The CUSUM test has long been used as a tool for detecting a change point, owing to its practical efficiency [40,41,42,43,44]. As regards the time series of counts, see [7,45,46,47,48,49,50].

Among the CUSUM tests, we adopted the residual-based CUSUM test, because the residual method can successfully discard the correlations of time series and enhance the performance of the CUSUM test in terms of both stability and power. See [51,52]. The authors of the recent reference [43,53] developed a simplistic residual-based CUSUM test for location-scale time series models, based on which the authors of [21,22] designated a hybridization of the SVR and CUSUM methods for handling the change point problem for AR and GARCH time series and demonstrated its superiority over classical models. However, their approach is not directly applicable and requires a new modification for effective performance, especially on the proxies used for the GARCH prediction, as seen in Section 3.4, as simple or exponential moving average type proxies conventionally used for SVR-GARCH models [22] would not work adequately in our current study. Here, we instead used the proxies obtained through the linear INGARCH fit to time series of counts.

The rest of this paper is organized as follows. Section 2 reviews the principle of the CUSUM test and CUSUM of squares test for the INGARCH models and then briefly describes how to apply the SVR-INGARCH method for constructing the CUSUM tests. Section 3 presents the SVR and TSVR-GARCH models for forecasting the conditional mean and describes the SVR and TSVR methods with PSO. Section 4 discusses the Monte Carlo simulations conducted to evaluate the performance of the proposed method. Section 5 discusses the performance of the real data analysis, using the return times of extreme events constructed based on the daily log-returns of Goldman Sachs (GS) stock prices. Finally, Section 6 provides concluding remarks.

## 2. INGARCH Model-Based Change Point Test

Let $\left\{Y_{t},t\geq 1\right\}$ be a time series of counts. In order to make inferences for $\left\{Y_{t}\right\}$, one can consider fitting a parametric model to $Y_{t}$, for instance, the INGARCH model with the conditional distribution of the one-parameter exponential family and the link function $f_{\theta }$, parameterized with $\theta \in \Theta \subset R^{d}$, that describes the conditional expectation, namely,

(1) $$\begin{array}{c} Y_{t}|F_{t-1}∼p\left(y|\eta_{t}\right),\;\;\;X_{t}:=E\left(Y_{t}|F_{t-1}\right)=f_{\theta }\left(X_{t-1},Y_{t-1}\right), \end{array}$$

where $F_{t}$ denotes the past information up to time *t*, $f_{\theta }$ is defined on $\left[0,\infty \right)\times N_{0}$ with $N_{0}=\left\{0,1,\ldots \right\}$, and p(·|·) is a probability mass function given by

$$\begin{array}{c} p(y|\eta )=\exp\{\eta y-A(\eta )\}h(y),\;\;\;\;y\geq 0, \end{array}$$

where η is the natural parameter, A(·) and h(·) are known real-valued functions, $B=A^{\prime }$ is strictly increasing, and $\eta_{t}=B^{-1}\left(X_{t}\right)$. $B(\eta_{t})$ and $B^{\prime }\left(\eta_{t}\right)$ are the conditional mean and variance of $Y_{t}$ over past observations, respectively. Symbols $X_{t}\left(\theta \right)$ and $\eta_{t}\left(\theta \right)$ are used to emphasize θ.

Conventionally, $f_{\theta }$ is assumed to be bounded below by some real number c>0 and to satisfy

(2) $$\begin{array}{c} \underset{\theta \in \Theta }{\sup}|f_{\theta }\left(x,y\right)-f_{\theta }\left(x^{\prime },y^{\prime }\right)|\leq \nu_{1}|x-x^{\prime }|+\nu_{2}\left|y-y^{\prime }\right| \end{array}$$

for all $x,x^{\prime }\geq 0$ and $y,y^{\prime }\in N_{0}$, where $\nu_{1},\nu_{2}\geq 0$ satisfies $\nu_{1}+\nu_{2}<1$, which, according to [12], allows $\left\{Y_{t}\right\}$ to be strictly stationary and ergodic, required for the consistency of the parameter estimates.

In practice, Poisson or negative binomial (NB) linear INGARCH(1,1) models with $X_{t}=\omega +\alpha X_{t-1}+\beta Y_{t-1}$, ω>0,α≥0,β≥0,α+β<1, are frequently used. For the former, we assume $Y_{t}|F_{t-1}∼\mathrm{Poisson}\left(X_{t}\right)$, whereas for the latter, we assume $Y_{t}|F_{t-1}∼NB\left(r,p_{t}\right),\;\;\;\;X_{t}=\frac{r(1-p_{t})}{p_{t}}=\omega +\alpha X_{t-1}+\beta Y_{t-1}$, where r∈N and Y∼ NB(r,p) denotes the negative binomial distribution with its mass function: $P\left(Y=k\right)=\frac{(k+r-1)!}{(r-1)!k!}{\left(1-p\right)}^{k}p^{r},$k≥0.

Let $\theta_{0}$ be a true parameter, which is assumed to be an interior point of the compact parameter space Θ. The $\theta_{0}$ is then estimated using the conditional likelihood function of model (1), based on the observations $Y_{1},\ldots ,Y_{n}$:

(3) $${\tilde{L}}_{n}\left(\theta \right)=\prod_{t=1}^{n}\exp\left\{\tilde{\eta_{t}}\left(\theta \right)Y_{t}-A\left(\tilde{\eta_{t}}\left(\theta \right)\right)\right\}h\left(Y_{t}\right),$$

where ${\tilde{\eta }}_{t}\left(\theta \right)=B^{-1}\left({\tilde{X}}_{t}\left(\theta \right)\right)$ is updated through the equations: ${\tilde{X}}_{t}\left(\theta \right)=f_{\theta }\left({\tilde{X}}_{t-1}\left(\theta \right),Y_{t-1}\right)$ for t≥2, ${\tilde{X}}_{1}\left(\theta \right)={\tilde{X}}_{1},$ with an initial value ${\tilde{X}}_{1}$.The conditional maximum likelihood estimator (CMLE) of $\theta_{0}$ is then obtained as the maximizer of the likelihood function in Equation (3):

$${\hat{\theta }}_{n}=\underset{\theta \in \Theta }{\mathrm{argmax}}{\tilde{L}}_{n}\left(\theta \right)=\underset{\theta \in \Theta }{\mathrm{argmax}}\sum_{t=1}^{n}{\tilde{\ell }}_{t}\left(\theta \right),$$

with ${\tilde{\ell }}_{t}\left(\theta \right)=\log p\left(Y_{t}|{\tilde{\eta }}_{t}\left(\theta \right)\right)={\tilde{\eta }}_{t}\left(\theta \right)Y_{t}-A\left({\tilde{\eta }}_{t}\left(\theta \right)\right)$. The authors of the reference [12,50] showed that, under certain conditions, ${\hat{\theta }}_{n}$ converges to $\theta_{0}$ in probability and $\sqrt{n}\left({\hat{\theta }}_{n}-\theta_{0}\right)$ is asymptotically normally distributed as *n* tends to *∞*. This ${\hat{\theta }}_{n}$ is harnessed to make prediction for calculating residuals.

In our current study, we aim to extend Model (1) to the nonparametric model:

(4) $$\begin{array}{c} Y_{t}|F_{t-1}∼p\left(y|\eta_{t}\right),\;\;\;X_{t}=g\left(X_{t-1},Y_{t-1}\right), \end{array}$$

where p(·|·) and *g* are unknown, and *g* is implicitly assumed to satisfy Equation (2). Provided $g\in \{f_{\theta };\theta \in \Theta \}$ and p(·|·) is known a priori, one can estimate *g* with $\hat{g}=f_{\hat{\theta }}$. Even if p(·|·) is unknown, one can still consider using the Poisson or NB quasi-likelihood estimator (QMLE) method as in [16]. See also [54] for various types of CUSUM tests based on the QMLEs. However, when one has no prior information as to *g*, the parametric modeling may hamper the inference, and in this case, one can estimate *g* with the nonparametric SVR method stated below in Section 3.

On Model (1), setting up the null and alternative hypotheses: $H_{0}:\theta$ remain the same over t=1,…,n. vs. $H_{1}:\mathrm{not}\;H_{0},$ The authors of the reference [50] considered the problem of detecting a change in θ based on the CUSUM test:

(5) $$\begin{array}{c} {\hat{T}}_{n}^{res}=\max_{1\leq k\leq n}\frac{1}{\sqrt{n}{\hat{\tau }}_{n}}|\sum_{t=1}^{k}{\hat{\epsilon }}_{t}-\frac{k}{n}\sum_{t=1}^{n}{\hat{\epsilon }}_{t}| \end{array}$$

with the residuals ${\hat{\epsilon }}_{t}=Y_{t}-{\tilde{X}}_{t}\left({\hat{\theta }}_{n}\right)$ and ${\hat{\tau }}_{n}^{2}=\frac{1}{n}\sum_{t=1}^{n}{\hat{\epsilon }}_{t}^{2}-{\left(\frac{1}{n}\sum_{t=1}^{n}{\hat{\epsilon }}_{t}\right)}^{2}.$ Furthermore, the authors of the references [55,56] employed the residual-based CUSUM of squares test:

(6) $$\begin{array}{c} {\hat{T}}_{n}^{square}=\max_{1\leq k\leq n}\frac{1}{\sqrt{n}{\tilde{\tau }}_{n}}|\sum_{t=1}^{k}{\hat{\epsilon }}_{t}^{2}-\frac{k}{n}\sum_{t=1}^{n}{\hat{\epsilon }}_{t}^{2}| \end{array}$$

with ${\tilde{\tau }}_{n}^{2}={\tilde{\gamma }}_{n}\left(0\right)+2\sum_{h=1}^{h_{n}}{\tilde{\gamma }}_{n}\left(h\right),{\tilde{\gamma }}_{n}\left(h\right)=\frac{1}{n}\sum_{t=1}^{n-h}\left({\hat{\epsilon }}_{t}^{2}-{\bar{\epsilon }}^{2}\right)\left({\hat{\epsilon }}_{t+h}^{2}-{\bar{\epsilon }}^{2}\right),$ ${\bar{\epsilon }}^{2}=\frac{1}{n}\sum_{t=1}^{n}{\hat{\epsilon }}_{t}^{2}$, and $h_{n}=\sqrt{2}{\left(\log_{10}n\right)}^{2}.$

The authors of the reference [50] verified that, under the null $H_{0}$, ${\hat{T}}_{n}^{res}$ behaves asymptotically the same as

$$\begin{array}{c} T_{n}=\max_{1\leq k\leq n}\frac{1}{n\tau }|\sum_{t=1}^{k}\epsilon_{t}-\frac{k}{n}\sum_{t=1}^{n}\epsilon_{t}|, \end{array}$$

where $\epsilon_{t}=Y_{t}-X_{t}\left(\theta_{0}\right)$ and $\tau^{2}=Var\left(\epsilon_{1}\right)$. As $\left\{\epsilon_{t}\right\}$ forms a sequence of martingale differences, we obtain $T_{n}\approx T:=\sup_{0\leq s\leq 1}\left|B^{\circ }\left(s\right)\right|$ in distribution [57], where $B^{\circ }$ denotes a Brownian bridge, owing to Donsker’s invariance principle, so that, as ${\hat{T}}_{n}^{res}\approx T_{n}$, we have ${\hat{T}}_{n}^{res}\approx T$ in distribution. For instance, $H_{0}$ is rejected if ${\hat{T}}_{n}^{res}\geq 1.3397$ at the level of 0.05, which is obtainable with Monte Carlo simulations. Similarly, the authors of the reference [55] verified that ${\hat{T}}_{n}^{square}\approx T$ in distribution, so that the same critical values as for the case of ${\hat{T}}_{n}^{res}$ can be harnessed. Provided that a change point exists, the location of change is identified as

$${\hat{k}}_{n}={\mathrm{argmax}}_{1\leq k\leq n}|\sum_{t=1}^{k}{\hat{\epsilon }}_{t}^{i}-\frac{k}{n}\sum_{t=1}^{n}{\hat{\epsilon }}_{t}^{i}|,\;i=1,2.$$

This CUSUM framework for parametric models can be easily adopted for nonparametric models as far as the residuals ${\hat{\epsilon }}_{t}$ can be accurately calculated, as seen in [21,22] who deal with the change point problem on SVR-ARMA and SVR-GARCH models. Below, when dealing with Model (4), instead of ${\hat{\epsilon }}_{t}$ in Equation (5), we use ${\hat{\epsilon }}_{t}=Y_{t}-\hat{g}\left(X_{t-1},Y_{t-1}\right)$ in the construction of the CUSUM tests in Equations (5) and (6).

When estimating *g* with SVR and TSVR, we train ${\left(y_{t},x_{t}\right)}^{T}$ either with $y_{t}={\tilde{X}}_{t}$ and $x_{t}={\left({\tilde{X}}_{t-1},Y_{t-1}\right)}^{T}$ or $y_{t}=Y_{t}$ and $x_{t}={\left({\tilde{X}}_{t-1},Y_{t-1}\right)}^{T}$, with some proper proxy ${\tilde{X}}_{t-1}$. The former has been used for the SVR-GARCH model in [21], while the latter is newly considered here, inspired by the fact that $Y_{t}=g\left(X_{t-1},Y_{t-1}\right)+\nu_{t}$, where the error process $\left\{\nu_{t}\right\}$ is a sequence of martingale differences, which also holds for Model (1) because we can express $Y_{t}=X_{t}+\nu_{t}$ in this case. See Step 3 in Section 4 below for more details.

## 3. SVR-INGARCH Model

In this section, we provide an outline of the SVR, TSVR, and PSO methods for a quick reference and describe the change point test based on the SVR-INGARCH model.

### 3.1. Support Vector Regression

SVR is an extension of the SVM, originally proposed by [58], and merits accurate nonlinear prediction. SVR aims to identify a nonlinear function of the form: $f\left(x\right)=w^{T}\phi \left(x\right)+b,$ where *x* denotes a vector of inputs, *w* and *b* are vectors of regression parameters, and ϕ is a known kernel function. The optimal *w* and *b* are determined from the ϵ-insensitive loss function (Vapnik, 2000):

(7) $$\begin{array}{c} \ell_{\epsilon }\left(y,f\left(x\right)\right)=\left\{\begin{array}{cc} |y-f(x)|-\epsilon & \mathrm{if}\;|y-f(x)|\geq \epsilon \\ 0 & \mathrm{otherwise}. \end{array}\right. \end{array}$$

Given input vectors $x_{i}$, scalar output $y_{i}$, i=1,…,n, and a constant C>0, we construct the objective function of the SVR as follows:

(8) $$\begin{array}{c} \mathrm{minimize}\;\frac{1}{2}{\left||w|\right|}^{2}+C\sum_{i=1}^{n}\left(\xi_{1,i}+\xi_{2,i}\right), \end{array}$$

$$\mathrm{subject}\;\mathrm{to}\;\left\{\begin{array}{c} y_{i}-w^{T}\phi \left(x_{i}\right)-b\leq \epsilon +\xi_{2,i}\\ w^{T}\phi \left(x_{i}\right)+b-y_{i}\leq \epsilon +\xi_{1,i}\\ \xi_{1,i}\geq 0,\;\xi_{2,i}\geq 0, \end{array}\right.$$

where $\xi_{1,i},\xi_{2,i}>0$ denote slack variables that allow some points to lie outside the ϵ-band with a penalty, and *C* denotes a trade-off between the function complexity and the training error.

To obtain the optimal *w* and *b*, we formulate an unconstrained optimization problem using Lagrange multipliers [27]. The Karush–Kuhn–Tucker (KKT) conditions then lead to the following dual form:

(9) $$\begin{array}{cc} \mathrm{maximize}\; & -\frac{1}{2}\sum_{i=1}^{n}\sum_{j=1}^{n}\left(\alpha_{1,i}-\alpha_{2,i}\right)\left(\alpha_{1,j}-\alpha_{2,j}\right)\phi {\left(x_{i}\right)}^{T}\phi \left(x_{j}\right)\\ \; & -\epsilon \sum_{i=1}^{n}\left(\alpha_{1,i}+\alpha_{2,i}\right)+\sum_{i=1}^{n}\left(\alpha_{1,i}-\alpha_{2,i}\right)y_{i}, \end{array}$$

subject to $\sum_{i=1}^{n}\left(\alpha_{1,i}-\alpha_{2,i}\right)=0,0\leq \alpha_{1,i}\leq C,\;0\leq \alpha_{2,i}\leq C$, where $\alpha_{1,i}$ and $\alpha_{2,i}$ denote dual variables [26]. Subsequently, the optimization problem in Equation (9) yields the solutions $\hat{w},\hat{b},\hat{f}$ of w,b,f, as follows:

$$\begin{array}{cc} \hat{w} & =\sum_{i=1}^{n}\left(\alpha_{1,i}-\alpha_{2,i}\right)\phi \left(x_{i}\right), \end{array}$$

(10) $$\begin{array}{cc} \hat{b} & =\left\{\begin{array}{cc} y_{i}-{\hat{w}}^{T}\phi \left(x_{i}\right)-\epsilon , & 0<\alpha_{1,i}<C\\ y_{i}-{\hat{w}}^{T}\phi \left(x_{i}\right)+\epsilon , & 0<\alpha_{2,i}<C, \end{array}\right.\\ \hat{f}\left(x\right) & =\sum_{i=1}^{n}\left(\alpha_{1,i}-\alpha_{2,i}\right)K\left(x_{i},x\right)+\hat{b} \end{array}$$

with $K\left(x,y\right)=\phi {\left(x\right)}^{T}\phi \left(y\right)$. In particular, we employ the Gaussian kernel for *K* in Equation (10), $K\left(x,y\right)=\exp\left(-\frac{||x-{y||}^{2}}{2\gamma^{2}}\right)$, and determine the tuning parameters $\gamma^{2}$, *C* in Equation (8), and ϵ in the loss function Equation (7) using the PSO method on the cube of $\left(C,\gamma^{2},\epsilon \right)$ with 1≤C≤100, $0.1\leq \gamma^{2}\leq 1$, and 0.1≤ϵ≤1.

### 3.2. Twin Support Vector Regression

TSVR is a modified version of SVR [30]. Similar to TSVM [59], TSVR derives two nonparallel planes $f_{1}\left(x\right)=w_{1}^{T}\phi_{1}\left(x\right)+b_{1}$ and $f_{2}\left(x\right)=w_{2}^{T}\phi_{2}\left(x\right)+b_{2}$, which respectively determines the $\epsilon_{1}$-sensitive downbound and $\epsilon_{2}$-sensitive upbound of data. Given input vectors $x_{i}$ and output $y_{i}$, i=1,…,n, the linear TSVR can be formulated as the constraint minimization problem as follows:

(11) $$\begin{array}{cc} \mathrm{minimize} & \;f\left(w_{1},b_{1},\xi_{1}\right)=\frac{1}{2}||Y-Aw_{1}-eb_{1}-e\epsilon_{1}{\left|\right|}^{2}+C_{1}e^{T}\xi_{1},\\ \mathrm{subject}\mathrm{to} & \;Y-Aw_{1}-eb_{1}\geq e\epsilon_{1}-\xi_{1}; \end{array}$$

(12) $$\begin{array}{cc} \mathrm{minimize} & \;f\left(w_{2},b_{2},\xi_{2}\right)=\frac{1}{2}||Y-Aw_{2}-eb_{2}+e\epsilon_{2}{\left|\right|}^{2}+C_{2}e^{T}\xi_{2},\\ \mathrm{subject}\mathrm{to} & \;Aw_{2}+eb_{2}-Y\geq e\epsilon_{2}-\xi_{2}, \end{array}$$

where $Y={\left(y_{1},\ldots ,y_{n}\right)}^{T}$, $A={\left(x_{1}\ldots x_{n}\right)}^{T},\;\epsilon_{1},\epsilon_{2}\geq$ 0, *e* denotes a vector whose components are all equal to 1, $C_{1},C_{2}\geq 0$ are hyperparameters, and $\xi_{1},\xi_{2}\geq 0$ are slack variables. Each QPP has *m* constraints instead of 2m constraints and has an advantage of faster computational speed. To obtain the optimal $w_{1}$ and $b_{1}$ in Equation (11), we solve the QPP using the Lagrangian function:

(13) $$\begin{array}{cc} L(w_{1},b_{1},\xi_{1},\alpha_{1},\beta_{1}):= & \;\frac{1}{2}||Y-Aw_{1}-eb_{1}-e\epsilon_{1}{\left|\right|}^{2}+C_{1}e^{T}\xi_{1}\\ \; & -\alpha_{1}^{T}\left(Y-Aw_{1}-eb_{1}-e\epsilon_{1}-\xi_{1}\right)-\beta_{1}^{T}\xi_{1}, \end{array}$$

where $\alpha_{1}\geq 0$ and $\beta_{1}$ are Lagrangian multiplier vectors. If there is an optimal solution, it must satisfy the following KKT conditions:

(14) $$\begin{array}{ccc} & & -A^{T}\left(Y-Aw_{1}-eb_{1}-e\epsilon_{1}\right)+A^{T}\alpha_{1}=0; \end{array}$$

(15) $$\begin{array}{ccc} & & -e^{T}\left(Y-Aw_{1}-eb_{1}=e\epsilon_{1}\right)+e^{T}\alpha_{1}=0;\\ & & C_{1}e-\alpha_{1}-\beta_{1}=0;\;\;\;\alpha_{1}^{T}\left(Y-Aw_{1}=eb_{1}-e\epsilon_{1}+\xi_{1}\right)=0,\;\alpha_{1}\geq 0;\\ & & \beta_{1}^{T}\xi_{1}=0,\;\beta_{1}\geq 0;\;\;\;Y-Aw_{1}+eb_{1}\geq e\epsilon_{1}-\xi_{1},\;\xi_{1}\geq 0;\\ & & Aw_{2}+eb_{2}-Y\geq e\epsilon_{2}-\xi_{2},\;\xi_{2}\geq 0. \end{array}$$

We define $G=\left(A\;e\right),h_{1}=Y-e\epsilon_{1}$, and $u_{1}={\left(w_{1}^{T}\;b_{1}\right)}^{T}$. Combining Equations (14) and (15), we have

(16) $$\begin{array}{c} u_{1}={\left(G^{T}G\right)}^{-1}G^{T}\left(h_{1}-\alpha_{1}\right). \end{array}$$

However, since $G^{T}G$ is only semidefinite, we introduce a regularization term σI, where σ>0 is very small, to overcome some ill-conditioned setting and use $u_{1}={\left(G^{T}G+\sigma I\right)}^{-1}G^{T}\left(h_{1}-\alpha_{1}\right)$. Next, substituting Equation (16) and the KKT conditions into Equation (13), we obtain the dual QPP form:

$$\begin{array}{cc} \mathrm{maximize} & \;-\frac{1}{2}\alpha_{1}^{T}G{\left(G^{T}G\right)}^{-1}G^{T}\alpha_{1}+h_{1}^{T}G{\left(G^{T}G\right)}^{-1}G^{T}\alpha_{1}-h_{1}^{T}\alpha_{1},\\ \mathrm{subject}\mathrm{to} & \;0\leq \alpha_{1}\leq C_{1}e, \end{array}$$

which yields $u_{1}$. Likewise, Equation (12) can acquire the dual QPP form:

$$\begin{array}{cc} \mathrm{minimize} & \;-\frac{1}{2}\alpha_{2}^{T}G{\left(G^{T}G\right)}^{-1}G^{T}\alpha_{2}-h_{2}^{T}G{\left(G^{T}G\right)}^{-1}G^{T}\alpha_{2}+h_{2}^{T}\alpha_{2},\\ \mathrm{subject}\mathrm{to} & \;0\leq \alpha_{2}\leq C_{2}e, \end{array}$$

where $\alpha_{2}$ is the Lagrangian multiplier vector and $h_{2}=Y+e\epsilon_{2}$. This yields $u_{2}={\left(w_{2}^{T}\;b_{2}\right)}^{T}$. Then, the estimated regressor can be formulated as follows:

$$f\left(x\right)=\frac{1}{2}\left(f_{1}\left(x\right)+f_{2}\left(x\right)\right)=\frac{1}{2}{\left(w_{1}+w_{2}\right)}^{T}x+\frac{1}{2}\left(b_{1}+b_{2}\right).$$

For extending the linear TSVR to a nonlinear one, we use the kernel-generated nonparallel hyperplanes, that is, $f_{1}\left(x\right)=K\left(x^{T},A^{T}\right)w_{1}+b_{1}$ and $f_{2}\left(x\right)=K\left(x^{T},A^{T}\right)w_{2}+b_{2}$. The optimization problem in this case is similar to the linear TSVR, and the nonlinear TSVR regressor is obtained as follows:

$$f\left(x\right)=\frac{1}{2}\left(f_{1}\left(x\right)+f_{2}\left(x\right)\right)=\frac{1}{2}{\left(w_{1}+w_{2}\right)}^{T}K\left(A,x\right)+\frac{1}{2}\left(b_{1}+b_{2}\right).$$

### 3.3. Particle Swarm Optimization Method

In a standard PSO algorithm [34], a set of *d* hyperparameters are considered as a particle of *d*-dimensional vector in search region $S=\{p={\left(p_{1},\ldots ,p_{d}\right)}^{T}\in R^{d};\;l_{k}\leq p_{k}\leq u_{k}\;\mathrm{with}\;l_{k},u_{k}\in R\;\mathrm{for}\;k=1,\ldots ,d\}.$ Here, *N* particles are modeled to move in *S*, with the position $p_{i}={\left(p_{i1},\ldots ,p_{id}\right)}^{T}$ and velocity $v_{i}={\left(v_{i1},\ldots ,v_{id}\right)}^{T}$ for i=1,…,N. The previous best position of the *i*-th particle is represented by $p_{i}^{best}={\left(p_{i1}^{best},\ldots ,p_{id}^{best}\right)}^{T}$, and the previous best position of all particles is represented by $g^{best}={\left(g_{1}^{best},\ldots ,g_{d}^{best}\right)}^{T}$. At each iteration *k*, where $1\leq k\leq K_{max}$ with maximum iteration number $K_{max}$, the velocity and position of the *i*-th particle are updated as follows:

$$\begin{array}{cc} v_{i}^{k+1} & =w_{k}v_{i}^{k}+c_{1}r_{1}\left(p_{i}^{best,k}-p_{i}^{k}\right)+c_{2}r_{2}\left(g^{best,k}-p_{i}^{k}\right),\\ p_{i}^{k+1} & =p_{i}^{k}+v_{i}^{k+1}, \end{array}$$

where $c_{1}$ and $c_{2}$ are two acceleration factors, $r_{1}$ and $r_{2}$ are two random variables following a uniform distribution over [0,1], and $w_{k}$ is an inertia factor defined by

$$\begin{array}{c} w_{k}=\left(w_{start}-w_{end}\right)\left(\frac{K_{max}-k}{K_{max}}\right)+w_{end}, \end{array}$$

where $w_{start}$ and $w_{end}$ are initial and final values of inertia. Since the positions of particles are updated, $p_{i}^{best}$ and $g^{best}$ are also updated as follows:

$$\begin{array}{cc} p_{i}^{best,k+1} & =\left\{\begin{array}{cc} p_{i}^{k+1}, & \mathrm{if}f\left(p_{i}^{k+1}\right)<f\left(p_{i}^{best,k}\right)\\ p_{i}^{best,k}, & \mathrm{otherwise} \end{array}\right.\\ g^{best,k+1} & ={\mathrm{argmin}}_{p_{i}^{best,k+1}}\;f\left(p_{i}^{best,k+1}\right). \end{array}$$

The finally updated $g^{best}$ in the above procedure is used as an optimal hyperparameter in estimating the SVR and TSVR models as seen below.

### 3.4. PSO-TSVR Model-Based CUSUM Test

In this subsection, we explain how to estimate $X_{t}$ in Model (4) using the SVR and TSVR methods with PSO as described above and how to construct the CUSUM test based on the residuals obtained from the SVR-INGARCH model. In the following steps, we assume that a time series $\left\{Y_{1},\ldots ,Y_{n},Y_{n+1},\ldots ,Y_{n+n^{\prime }}\right\}$ has no change points.

- **Step 1.** As in Section 3.3, in order to apply the PSO method, we set a space of hyperparameters and initialize the positions $\left\{p_{1}^{0},\ldots ,p_{N}^{0}\right\}$ and velocities $\left\{v_{1}^{0},\ldots ,v_{N}^{0}\right\}$ of particles to be evaluated within this space. Subsequently, we divide the given time series into two groups of time series, $\left\{Y_{1},\ldots ,Y_{n}\right\}$ and $\left\{Y_{n+1},\ldots ,Y_{n+n^{\prime }}\right\}$. The former is used as a training set while the latter is used as a validation set.
- **Step 2.** Compute the initial estimates of $X_{t}$ based on the training time series. For this task, we use two different methods. The first method is using moving averages (Niemira, 1994):
  (17) $$\begin{array}{cc} {\tilde{X}}_{t} & =\frac{1}{m}\sum_{j=1}^{m}Y_{t-j+1}, \end{array}$$
  where *m* is a positive integer. When *t* is smaller than *m*, ${\tilde{X}}_{t}$ is computed as an average of the first to the *t*-th squares of observations, i.e. ${\tilde{X}}_{t}=\sum_{j=1}^{t}Y_{t-j+1}/t$ when t<m.
  The second method is using the Poisson QMLE [54] assuming that the time series follows Model (1), for example,
  (18) $$\begin{array}{cc} {\tilde{X}}_{t} & =\hat{\omega }+\hat{\alpha }{\tilde{X}}_{t-1}+\hat{\beta }Y_{t-1}. \end{array}$$
  These estimates play the role of proxy of $X_{t}$ and replace the true conditional volatility.
- **Step 3.** For particles $p_{i}^{k}$, $k=1,2,\ldots K_{max}$, we train ${\left(y_{t},x_{t}\right)}^{T}$, either with $y_{t}={\tilde{X}}_{t}$ and $x_{t}={\left({\tilde{X}}_{t-1},Y_{t-1}\right)}^{T}$ or $y_{t}=Y_{t}$ and $x_{t}={\left({\tilde{X}}_{t-1},Y_{t-1}\right)}^{T}$ with proxy ${\tilde{X}}_{t-1}$ to the SVR and TSVR models to obtain $\hat{g}$. Subsequently, for the first, we obtain
  (19) $$\begin{array}{c} {\hat{X}}_{t}=\hat{g}\left(Y_{t-1},{\tilde{X}}_{t-1}\right), \end{array}$$
  named “${\hat{X}}_{t}$-targeting”, and for the second,
  (20) $$\begin{array}{c} {\hat{Y}}_{t}=\hat{g}\left(Y_{t-1},{\tilde{X}}_{t-1}\right), \end{array}$$
  named “${\hat{Y}}_{t}$-targeting”, where ${\hat{Y}}_{t}$ is an estimate of $Y_{t}$, which is an estimate $X_{t}$ as well since ${\hat{Y}}_{t}$ itself is the conditional expectation.
- **Step 4.** Applying the estimated SVR and TSVR models and using the same proxy formula as in **Step 2** for the validation time series, the mean absolute error (MAE) is computed as follows:
  $$\begin{array}{c} \mathrm{MAE}=\frac{1}{n^{\prime }}\sum_{t=n+1}^{n+n^{{}^{\prime }}}\left|{\hat{X}}_{t}-{\tilde{X}}_{t}\right| \end{array}$$
  for the case of Equation (19), and
  $$\begin{array}{c} \mathrm{MAE}=\frac{1}{n^{\prime }}\sum_{t=n+1}^{n+n^{{}^{\prime }}}\left|{\hat{Y}}_{t}-{\tilde{X}}_{t}\right| \end{array}$$
  for the case of Equation (20). The MAE is employed here because it is more robust against outliers in a model fitting than the root mean square error.
- **Step 5.** Update the $p_{i}^{k},v_{i}^{k},p_{i}^{best,k}$, and $g^{best,k}$ as in Section 3.3 and repeat **Steps 3 and 4** until the MAE in **Step 4** converges within a limit or *k* reaches the maximum iteration number $K_{max}$.
- **Step 6.** Apply the estimated SVR and TSVR models with selected parameters in **Step 5** to a testing time series to perform the CUSUM tests in Equations (5) and (6) based on the residuals ${\hat{\epsilon }}_{t}=Y_{t}-\hat{g}\left(Y_{t-1},{\tilde{X}}_{t-1}\right)$.

## 4. Simulation Results

In this section, we apply the PSO-SVR and -TSVR models to the INAR(1) and INGARCH(1,1) models, and evaluate the performance of the proposed CUSUM tests. For this task, we generate a time series of length 1000 ($n=500,n^{\prime }=500)$ to evaluate the empirical sizes and powers at the nominal level of 0.05. The size and power are calculated as the rejection number of the null hypothesis of no changes out of 500 repetitions. The simulations were conducted with R version 3.6.3, running on Windows 10. Moreover, we use the following R packages: “pso” for the PSO [60], “kernlab” for the Gaussian kernel [61], and “osqp” [62] for solving the quadratic problem. The procedure for the simulation is as follows.

- **Step 1.** Generate a time series of length n=1000 to train the PSO-SVR and -TSVR models.
- **Step 2.** Apply the estimation scheme described in Section 4. For the proxy of moving averages, we used m=5. In this procedure, we divide the given time series into n=500 and $n^{\prime }=500$ in **Step 1** of Section 4.
- **Step 3.** Generate a testing time series of length n=1000 to evaluate the size and power. For computing sizes, we generate a time series of no changes, whereas to examine the power, we generate a time series with a change point in the middle.
- **Step 4.** Apply the estimated model in **Step 2** to the time series of **Step 3** and conduct the residual CUSUM and CUSUM of squares tests.
- **Step 5.** Repeat the above steps *N* times, e.g., 500, and then compute the empirical sizes and powers.

We consider the INGARCH(1,1) and INAR(1) models, as these are the most acclaimed models in practice:

- **Model 1**. $Y_{t}|F_{t-1}∼\mathrm{Poisson}\left(X_{t}\right),\;X_{t}=\omega +\alpha X_{t-1}+\beta Y_{t-1},$
- **Model 2**. $Y_{t}=\phi \circ Y_{t-1}+Z_{t},\;Z_{t}∼\mathrm{Poisson}\left(\omega \right)$, where ∘ is a binomial thinning operator and |ϕ|<1.

Further, upon one referee’s suggestion, we also consider the softplus INGARCH(1,1) model in [63]:

- **Model 3**. $Y_{t}|F_{t-1}∼\mathrm{Poisson}\left(X_{t}\right),\;X_{t}=s_{c}\left(\omega +\alpha Y_{t-1}+\beta X_{t-1}\right)$, where $s_{c}\left(x\right)=c\log\left(1+\exp\left(x/c\right)\right)$.

Under the null hypothesis, we use the parameter settings as follows.

- Model 1:
  − Case 1: ω=3,α=0.3,β=0.3;
  − Case 2: ω=5,α=0.3,β=0.3;
  − Case 3: ω=3,α=0.6,β=0.3;
  − Case 4: ω=3,α=0.3,β=0.6;
- Model 2:
  − Case 1: ω=3,ϕ=0.3;
  − Case 2: ω=5,ϕ=0.3;
  − Case 3: ω=3,ϕ=0.7;
- Model 3:
  − c=1,ω=3,α=0.3,β=0.3.

Under the alternative hypothesis, we only consider the case of one parameter change, while the other parameters remain the same. Table A1, Table A2, Table A3 and Table A4 in Appendix A summarize the results for Model 1. Here, MA and ML denote the proxies obtained from the moving average in Equation (17) and Poisson QMLE in Equation (18), and ${\hat{Y}}_{t}$ and ${\hat{X}}_{t}$, respectively, denote the two targeting methods in Equation (20) and Equation (19). The tables show that the difference between the SVR and TSVR methods is marginal. Moreover, in most cases, ${\hat{T}}_{n}^{square}$ appears to be much more stable than ${\hat{T}}_{n}^{res}$; that is, the latter test suffers from more severe size distortions. In terms of power, ${\hat{T}}_{n}^{res}$ with the ML proxy and ${\hat{X}}_{t}$-targeting tends to outperform the others. However, the gap between this test and ${\hat{T}}_{n}^{square}$ is only marginal; therefore, considering the stability of the test, ${\hat{T}}_{n}^{square}$ is highly favored for Model 1. Table A5, Table A6 and Table A7 summarize the result for Model 2, showing that ${\hat{T}}_{n}^{res}$ exhibits a more stable performance for the INAR models than for the INGARCH models. However, it is still not as stable as ${\hat{T}}_{n}^{square}$ and tends to outperform ${\hat{T}}_{n}^{square}$ in terms of power. Table A8 summarizes the result for Model 3, showing no significant differences from the results of the previous models. This result, to a certain extent, coincides with that of Lee and Lee (2020) who considered parametric INGARCH models for a change point test. Overall, our findings strongly confirm the reliability of using ${\hat{T}}_{n}^{square}$, particularly with the ML proxy and ${\hat{X}}_{t}$-targeting. However, in practice, one can additionally implement ${\hat{T}}_{n}^{res}$ because either test can react more sensitively to a specific situation in comparison to the other.

Table A9 lists the computing times (in seconds) of the SVR and TSVR methods when implemented in R on Windows 10, running on a PC with an Intel i7-3770 processor (3.4 GHz) with 8 GB of RAM, wherein the figures denote the averages of training times in simulations, and the values in the parentheses indicate the sample standard deviations. In each model and parameter setting, the values of the two quickest results are written in boldface. As reported by [21,30], the TSVR method is shown to markedly reduce the CPU time. In particular, the results indicate that the computational speed of the TSVR-based method, with the ML proxy and ${\hat{X}}_{t}$-targeting, appears to be the fastest in most cases. The result suggests that using the TSVR-based CUSUM tests is beneficial when computational speed is of significance to the implementation.

## 5. Real Data Analysis

**Figure 1:**
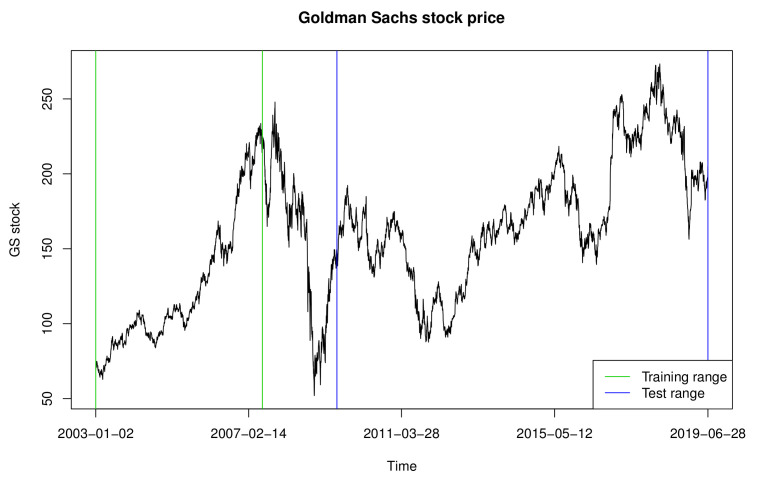
Goldman Sachs stock price.

**Figure 2:**
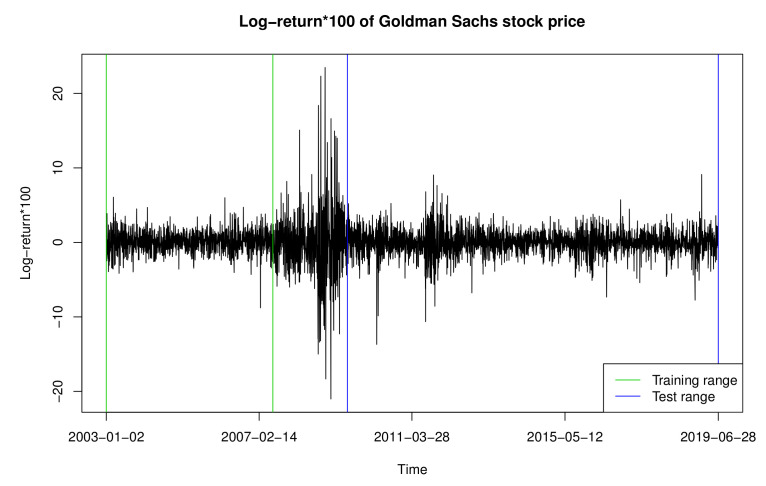
Log-return of Goldman Sachs stock price.

In this section, we analyze the return times of extreme events constructed based on the daily log-returns of GS stock prices from 1 January 2003, to 28 June 2019, obtained using the R package “quantmod.” We used data from 2 January 2003, to 29 June 2007, as the training set and that from 1 July 2009, to 28 June 2019, as the test set. Figure 1 and Figure 2 exhibit the GS stock prices and 100 times the log-returns, with their ranges denoted by the green and blue vertical lines, respectively. As shown in Figure 2, the time series between the training and test sets has severe volatility, owing to the financial crisis that occurred in 2008; therefore, it is omitted from our data analysis.

Before applying the PSO-SVR-INGARCH and PSO-TSVR-INGARCH methods, similarly to [12,14], we first transform the given time series into the hitting times $\tau_{1},\tau_{2},\ldots$, for which the log-returns of the GS stock fall outside the 0.15 and 0.85 quantiles of the training data, that is, -1.242 and 1.440, respectively. More specifically, $\tau_{1}=\inf\left\{t\geq 1;w_{t}\notin \left[-1.242,1.440\right]\right\}$, $\tau_{2}=\inf\left\{t\geq \tau_{1};w_{t}\notin \left[-1.242,1.440\right]\right\},\ldots$, where $w_{t}$ denote the 100 times log-returns. We then set $Y_{t}:=\tau_{t}-\tau_{t-1}$, which forms the return times of these extreme events. Consequently, the training set is transformed into an integer-valued time series of length 341, and the test set is transformed into that of length 844 (see Figure 2), plotting $Y_{t}$.

**Figure 3:**
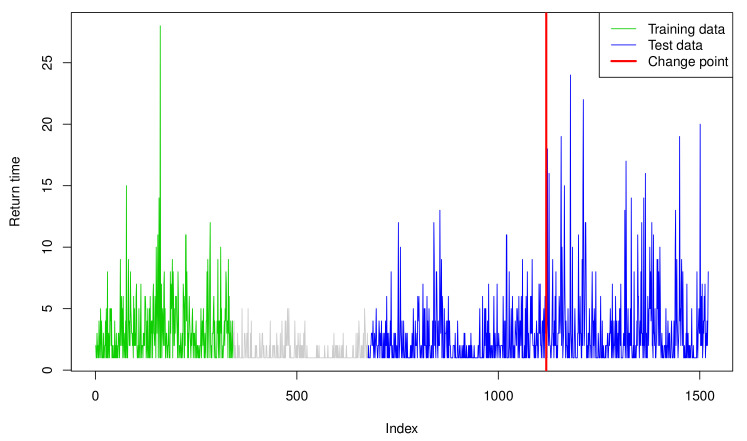
Return time of extreme events of Goldman Sachs stock prices.

To determine whether the training set exhibits change, we apply the Poisson QMLE method and CUSUM of squares test from [54]. The result shows that the CUSUM statistics ${\hat{T}}_{n}^{square}$ has a value of 0.590, which is smaller than the theoretical critical value of 1.358; thus, the null hypothesis of no change is not rejected at the nominal level of 0.05, supporting the adequacy of the training set. The residual-based CUSUM of squares tests based on the SVR and TSVR models with the ML proxy and ${\hat{X}}_{t}$-targeting are then applied; subsequently, both tests detect a change point at the 441st observation of the testing data, corresponding to 16 October 2013. The red vertical line in Figure 3 denotes the detected change point.

To examine how the change affects the dynamic structure of the time series, we fit a Poisson linear INGARCH model to the training and testing time series before and after the change point. For the training time series, the fitted INGARCH model appears to have $\hat{\omega }=0.334,\hat{\alpha }=0.813$, and $\hat{\beta }=0.086$. Conversely, for the testing time series before the change, we obtain $\hat{\omega }=0.135,\hat{\alpha }=0.878$, and $\hat{\beta }=0.067$, which are not as different as those from the training time series case. However, after the change point, the fitted parameters are shown to be $\hat{\omega }=1.045,\hat{\alpha }=0.560$, and $\hat{\beta }=0.147$, thus confirming a significant change in the parameters. For instance, the sum of α and β in the training data is 0.899, which changes from 0.945 to 0.707 in the testing data.

## 6. Concluding Remarks

In this study, we proposed the CUSUM test based on the residuals obtained with the SVR and TSVR-INGARCH models to detect a parameter change in the conditional mean of the time series of counts. To improve accuracy and efficiency, we also employed the PSO method to obtain an optimal set of hyperparameters. Monte Carlo simulations were conducted using the INAR and INGARCH models with various parameter settings. The results showed that the TSVR method using the ML proxy and the conditional mean ${\hat{X}}_{t}$-targeting method is recommendable, as it generally performs well and markedly reduces computational time. Our method was then applied to the analysis of the return times of extreme events constructed based on the daily log-returns of Goldman Sachs stock prices and, subsequently, detected one change. Overall, our findings, based on a simulation study and real data analysis, demonstrated the validity of our method. Although the proposed method performs well in general, it might have a limitation in its performance when the amount of available training data is not large enough or the dataset has features that can violate the stationarity, e.g., high volatilities. The method can also suffer from over-fitting to a specific training sample. Thus, it would be an important task to develop more robust methods, which we leave as our future project.

## Data Availability

Publicly available datasets were analyzed in this study. This data can be found here: https://finance.yahoo.com, (accessed on 10 August 2020).

## Abbreviations

The following abbreviations are used in this manuscript:

SVR	Support vector regression
TSVR	Twin support vector regression
PSO	Particle swarm optimization
INGARCH	Integer-valued generalized autoregressive conditional heteroskedasticity
CUSUM	Cumulative sum
INAR	Integer-valued autoregressive
QMLE	Quasi-maximum likelihood estimation
ARMA	Autoregressive and moving average
GARCH	Generalized autoregressive conditional heteroskedasticity
NB	Negative binomial
CMLE	Conditional maximum likelihood estimator
SVM	Support vector machine
MAE	Mean absolute error

## Appendix A

**Table A1:** Empirical sizes and powers for the INGARCH(1,1) model, Case 1.

ω=3		Method	SVR				TSVR			
α=0.3		Target	${\hat{Y}}_{t}$		${\hat{X}}_{t}$		${\hat{Y}}_{t}$		${\hat{X}}_{t}$	
β=0.3		Proxy	MA	ML	MA	ML	MA	ML	MA	ML
size		${\hat{T}}_{n}^{res}$	0.072	0.104	0.000	0.096	0.076	0.064	0.000	0.074
		${\hat{T}}_{n}^{square}$	0.040	0.034	0.032	0.042	0.036	0.038	0.032	0.046
power	ω→5	${\hat{T}}_{n}^{res}$	1.000	1.000	0.640	1.000	0.984	0.860	0.274	0.996
		${\hat{T}}_{n}^{square}$	0.968	0.980	0.776	0.994	0.984	0.964	0.808	0.990
	ω→10	${\hat{T}}_{n}^{res}$	1.000	1.000	1.000	1.000	0.972	0.962	0.948	0.956
		${\hat{T}}_{n}^{square}$	1.000	1.000	1.000	1.000	1.000	1.000	1.000	1.000
	α→0.5	${\hat{T}}_{n}^{res}$	1.000	1.000	0.978	1.000	0.976	0.868	0.620	0.982
		${\hat{T}}_{n}^{square}$	0.986	0.994	0.936	0.944	0.986	0.992	0.944	1.000
	β→0.5	${\hat{T}}_{n}^{res}$	1.000	1.000	0.988	1.000	0.982	0.870	0.664	0.982
		${\hat{T}}_{n}^{square}$	0.952	0.950	0.826	0.956	0.916	0.924	0.826	0.980

**Table A2:** Empirical sizes and powers for the INGARCH(1,1) model, Case 2.

ω=5		Method	SVR				TSVR			
α=0.3		Target	${\hat{Y}}_{t}$		${\hat{X}}_{t}$		${\hat{Y}}_{t}$		${\hat{X}}_{t}$	
β=0.3		Proxy	MA	ML	MA	ML	MA	ML	MA	ML
size		${\hat{T}}_{n}^{res}$	0.062	0.114	0.000	0.104	0.072	0.068	0.000	0.084
		${\hat{T}}_{n}^{square}$	0.048	0.044	0.048	0.044	0.038	0.048	0.050	0.046
power	ω→3	${\hat{T}}_{n}^{res}$	1.000	1.000	0.878	1.000	1.000	0.986	0.826	1.000
		${\hat{T}}_{n}^{square}$	0.620	0.558	0.636	0.566	0.556	0.520	0.590	0.504
	ω→10	${\hat{T}}_{n}^{res}$	1.000	1.000	1.000	1.000	0.962	0.920	0.770	1.000
		${\hat{T}}_{n}^{square}$	0.996	0.996	0.996	1.000	0.992	1.000	0.986	1.000
	α→0.5	${\hat{T}}_{n}^{res}$	1.000	1.000	1.000	1.000	0.974	0.918	0.790	1.000
		${\hat{T}}_{n}^{square}$	0.996	0.994	0.988	1.000	0.986	0.980	0.956	1.000
	β→0.5	${\hat{T}}_{n}^{res}$	1.000	1.000	1.000	1.000	0.976	0.928	0.824	1.000
		${\hat{T}}_{n}^{square}$	0.990	0.988	0.902	0.994	0.946	0.948	0.870	1.000

**Table A3:** Empirical sizes and powers for the INGARCH(1,1) model, Case 3.

ω=3		Method	SVR				TSVR			
α=0.6		Target	${\hat{Y}}_{t}$		${\hat{X}}_{t}$		${\hat{Y}}_{t}$		${\hat{X}}_{t}$	
β=0.3		Proxy	MA	ML	MA	ML	MA	ML	MA	ML
size		${\hat{T}}_{n}^{res}$	0.176	0.278	0.002	0.230	0.154	0.180	0.000	0.180
		${\hat{T}}_{n}^{square}$	0.036	0.036	0.042	0.046	0.032	0.048	0.040	0.042
power	ω→5	${\hat{T}}_{n}^{res}$	1.000	1.000	0.990	1.000	0.970	0.928	0.746	1.000
		${\hat{T}}_{n}^{square}$	0.964	0.948	0.856	0.948	0.956	0.956	0.866	0.972
	α→0.3	${\hat{T}}_{n}^{res}$	1.000	1.000	1.000	1.000	0.998	0.996	1.000	1.000
		${\hat{T}}_{n}^{square}$	0.998	0.998	1.000	0.998	0.994	0.972	0.996	1.000
	β→0.1	${\hat{T}}_{n}^{res}$	1.000	1.000	1.000	1.000	1.000	1.000	1.000	1.000
		${\hat{T}}_{n}^{square}$	0.992	0.998	0.974	0.998	0.958	0.908	0.860	0.998

**Table A4:** Empirical sizes and powers for the INGARCH(1,1) model, Case 4.

ω=3		Method	SVR				TSVR			
α=0.3		Target	${\hat{Y}}_{t}$		${\hat{X}}_{t}$		${\hat{Y}}_{t}$		${\hat{X}}_{t}$	
β=0.6		Proxy	MA	ML	MA	ML	MA	ML	MA	ML
size		${\hat{T}}_{n}^{res}$	0.258	0.260	0.020	0.174	0.172	0.158	0.018	0.100
		${\hat{T}}_{n}^{square}$	0.036	0.040	0.032	0.030	0.032	0.026	0.024	0.030
power	ω→5	${\hat{T}}_{n}^{res}$	0.992	0.996	0.820	0.998	0.934	0.904	0.422	0.848
		${\hat{T}}_{n}^{square}$	0.492	0.472	0.326	0.428	0.602	0.586	0.570	0.776
	α→0.1	${\hat{T}}_{n}^{res}$	1.000	1.000	0.994	1.000	0.996	0.994	0.994	1.000
		${\hat{T}}_{n}^{square}$	0.800	0.780	0.436	0.604	0.634	0.674	0.406	0.644
	β→0.3	${\hat{T}}_{n}^{res}$	1.000	1.000	0.996	1.000	0.998	0.994	1.000	1.000
		${\hat{T}}_{n}^{square}$	0.926	0.920	0.716	0.862	0.882	0.890	0.712	0.956

**Table A5:** Empirical sizes and powers for the INAR(1) model, Case 1.

ω=3 ϕ=0.3		Method	SVR				TSVR			
		Target	${\hat{Y}}_{t}$		${\hat{X}}_{t}$		${\hat{Y}}_{t}$		${\hat{X}}_{t}$	
		Proxy	MA	ML	MA	ML	MA	ML	MA	ML
size		${\hat{T}}_{n}^{res}$	0.052	0.070	0.000	0.070	0.064	0.060	0.000	0.060
		${\hat{T}}_{n}^{square}$	0.048	0.060	0.064	0.054	0.066	0.052	0.058	0.062
power	ω→5	${\hat{T}}_{n}^{res}$	1.000	1.000	0.466	1.000	0.996	0.990	0.156	1.000
		${\hat{T}}_{n}^{square}$	0.960	0.994	0.800	1.000	0.982	0.990	0.810	1.000
	ω→10	${\hat{T}}_{n}^{res}$	1.000	1.000	1.000	1.000	0.984	0.966	0.838	0.976
		${\hat{T}}_{n}^{square}$	1.000	1.000	1.000	1.000	1.000	1.000	1.000	1.000
	ϕ→0.1	${\hat{T}}_{n}^{res}$	0.906	0.932	0.000	0.940	0.922	0.922	0.000	0.934
		${\hat{T}}_{n}^{square}$	0.218	0.150	0.166	0.080	0.084	0.078	0.178	0.072
	ϕ→0.5	${\hat{T}}_{n}^{res}$	1.000	1.000	0.106	1.000	0.994	0.992	0.026	1.000
		${\hat{T}}_{n}^{square}$	0.600	0.598	0.164	0.584	0.606	0.562	0.186	0.542

**Table A6:** Empirical sizes and powers for the INAR(1) model, Case 2.

ω=5		Method	SVR				TSVR			
ϕ=0.3		Target	${\hat{Y}}_{t}$		${\hat{X}}_{t}$		${\hat{Y}}_{t}$		${\hat{X}}_{t}$	
		Proxy	MA	ML	MA	ML	MA	ML	MA	ML
size		${\hat{T}}_{n}^{res}$	0.068	0.084	0.000	0.070	0.068	0.068	0.000	0.060
		${\hat{T}}_{n}^{square}$	0.062	0.044	0.038	0.048	0.042	0.048	0.036	0.040
power	ω→3	${\hat{T}}_{n}^{res}$	1.000	1.000	0.630	1.000	1.000	0.998	0.692	1.000
		${\hat{T}}_{n}^{square}$	0.578	0.566	0.716	0.442	0.492	0.552	0.708	0.452
	ω→10	${\hat{T}}_{n}^{res}$	1.000	1.000	0.992	1.000	0.976	0.962	0.688	1.000
		${\hat{T}}_{n}^{square}$	0.986	0.998	0.996	1.000	1.000	1.000	0.998	1.000
	ϕ→0.1	${\hat{T}}_{n}^{res}$	0.990	1.000	0.002	0.996	0.998	0.996	0.000	0.996
		${\hat{T}}_{n}^{square}$	0.282	0.224	0.162	0.096	0.122	0.148	0.170	0.112
	ϕ→0.5	${\hat{T}}_{n}^{res}$	1.000	1.000	0.340	1.000	1.000	0.996	0.138	1.000
		${\hat{T}}_{n}^{square}$	0.800	0.822	0.172	0.830	0.812	0.774	0.204	0.812

**Table A7:** Empirical sizes and powers for the INAR(1) model, Case 3.

ω=3 ϕ=0.7		Method	SVR				TSVR			
		Target	${\hat{Y}}_{t}$		${\hat{X}}_{t}$		${\hat{Y}}_{t}$		${\hat{X}}_{t}$	
		Proxy	MA	ML	MA	ML	MA	ML	MA	ML
size		${\hat{T}}_{n}^{res}$	0.116	0.072	0.002	0.054	0.092	0.056	0.000	0.048
		${\hat{T}}_{n}^{square}$	0.048	0.058	0.034	0.054	0.054	0.052	0.038	0.058
power	ω→5	${\hat{T}}_{n}^{res}$	1.000	1.000	0.762	1.000	0.978	0.916	0.426	0.976
		${\hat{T}}_{n}^{square}$	0.940	0.960	0.648	0.942	0.940	0.936	0.728	0.990
	ϕ→0.3	${\hat{T}}_{n}^{res}$	1.000	1.000	0.896	1.000	1.000	1.000	0.946	1.000
		${\hat{T}}_{n}^{square}$	0.852	0.846	0.284	0.836	0.854	0.906	0.210	0.898

**Table A8:** Empirical sizes and powers for the softplus INGARCH(1,1) model.

c=1,ω=3 α=0.3,β=0.3		Method	SVR				TSVR			
		Target	${\hat{Y}}_{t}$		${\hat{X}}_{t}$		${\hat{Y}}_{t}$		${\hat{X}}_{t}$	
		Proxy	MA	ML	MA	ML	MA	ML	MA	ML
size		${\hat{T}}_{n}^{res}$	0.072	0.114	0.000	0.108	0.080	0.062	0.000	0.072
		${\hat{T}}_{n}^{square}$	0.034	0.032	0.034	0.038	0.032	0.036	0.030	0.046
power	ω→5	${\hat{T}}_{n}^{res}$	1.000	0.998	0.666	1.000	0.978	0.862	0.284	0.996
		${\hat{T}}_{n}^{square}$	0.966	0.982	0.792	0.992	0.984	0.962	0.828	0.986
	α→0.5	${\hat{T}}_{n}^{res}$	1.000	0.998	0.984	1.000	0.980	0.834	0.658	0.982
		${\hat{T}}_{n}^{square}$	0.982	0.986	0.932	0.994	0.982	0.988	0.924	1.000
	β→0.5	${\hat{T}}_{n}^{res}$	1.000	1.000	0.984	1.000	0.980	0.842	0.704	0.978
		${\hat{T}}_{n}^{square}$	0.948	0.938	0.810	0.950	0.926	0.936	0.830	0.964

**Table A9:** Computing times for training the SVR and the TSVR methods.

Method		SVR				TSVR			
Target		${\hat{Y}}_{t}$		${\hat{X}}_{t}$		${\hat{Y}}_{t}$		${\hat{X}}_{t}$	
Proxy		MA	ML	MA	ML	MA	ML	MA	ML
INGARCH	ω=3,	918.02	863.33	1466.45	825.02	193.70	**192.68**	**185.44**	193.09
	α=0.3,β=0.3	(190.61)	(201.94)	(247.97)	(305.41)	(19.79)	(19.11)	(15.94)	(21.02)
	ω=5,	1633.97	1065.13	1231.81	1029.88	224.41	245.24	**155.52**	**162.26**
	α=0.3,β=0.3	(319.64)	(229.89)	(191.73)	(363.53)	(22.42)	(27.33)	(16.06)	(17.89)
	ω=3,	1979.82	1552.36	2456.07	1766.37	195.97	207.49	**191.79**	**192.45**
	α=0.6,β=0.3	(242.94)	(366.01)	(395.27)	(666.71)	(20.59)	(21.05)	(19.60)	(19.34)
	ω=3,	1877.27	1940.01	2697.95	2219.46	1198.25	196.93	**186.23**	**189.34**
	α=0.3,β=0.6	(389.79)	(347.92)	(356.38)	(587.99)	(20.95)	(20.56)	(18.98)	(21.95)
INAR	ω=3,ϕ=0.3	780.01	1379.59	1340.27	754.08	191.38	**189.67**	**182.64**	192.35
		(189.10)	(358.28)	(256.68)	(255.29)	(20.17)	(19.59)	(16.42)	(26.07)
	ω=5,ϕ=0.3	1000.50	972.17	1128.04	1336.76	240.72	226.47	**150.59**	**105.45**
		(235.13)	(240.82)	(217.57)	(516.75)	(31.46)	(23.05)	(12.85)	(14.81)
	ω=10,ϕ=0.3	828.82	1437.23	1542.48	769.35	194.25	193.05	**184.82**	**194.41**
		(208.43)	(378.40)	(239.95)	(288.29)	(19.67)	(17.84)	(16.14)	(21.17)
softplus	c=1,ω=3	692.41	711.94	911.11	692.71	193.99	**193.54**	**186.39**	196.66
INGARCH	α=0.3,β=0.3	(118.22)	(146.43)	(157.28)	(206.60)	(19.01)	(18.86)	(17.34)	(20.83)
